# A Validated Model of Serum Anti-Müllerian Hormone from Conception to Menopause

**DOI:** 10.1371/journal.pone.0022024

**Published:** 2011-07-15

**Authors:** Thomas W. Kelsey, Phoebe Wright, Scott M. Nelson, Richard A. Anderson, W. Hamish B Wallace

**Affiliations:** 1 School of Computer Science, University of St. Andrews, St. Andrews, Scotland, United Kingdom; 2 University of Edinburgh, Edinburgh, Scotland, United Kingdom; 3 Centre for Population and Health Sciences, University of Glasgow, Glasgow, Scotland, United Kingdom; 4 MRC Centre for Reproductive Health, University of Edinburgh, Edinburgh, Scotland, United Kingdom; 5 Division of Reproductive and Developmental Sciences, University of Edinburgh, Edinburgh, Scotland, United Kingdom; Indiana University, United States of America

## Abstract

**Background:**

Anti-Müllerian hormone (AMH) is a product of growing ovarian follicles. The concentration of AMH in blood may also reflect the non-growing follicle (NGF) population, i.e. the ovarian reserve, and be of value in predicting reproductive lifespan. A full description of AMH production up to the menopause has not been previously reported.

**Methodology/Principal Findings:**

By searching the published literature for AMH concentrations in healthy pre-menopausal females, and using our own data (combined 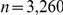) we have generated and robustly validated the first model of AMH concentration from conception to menopause. This model shows that 34% of the variation in AMH is due to age alone. We have shown that AMH peaks at age 24.5 years, followed by a decline to the menopause. We have also shown that there is a neonatal peak and a potential pre-pubertal peak. Our model allows us to generate normative data at all ages.

**Conclusions/Significance:**

These data highlight key inflection points in ovarian follicle dynamics. This first validated model of circulating AMH in healthy females describes a transition period in early adulthood, after which AMH reflects the progressive loss of the NGF pool. The existence of a neonatal increase in gonadal activity is confirmed for females. An improved understanding of the relationship between circulating AMH and age will lead to more accurate assessment of ovarian reserve for the individual woman.

## Introduction

The human ovary establishes its complete complement of primordial follicles during fetal life, with recruitment and thereby depletion of this dormant primordial follicle pool required for normal fertility but ultimately leading to reproductive senescence [1]. Primordial follicles are recruited continuously from before birth to join the early growing cohort (initial recruitment). After puberty, at every new cycle a limited number of follicles are recruited from this cohort of small growing follicles (cyclic recruitment), followed by a final selection for dominance and ovulation of a single follicle [2], [3]. Thus, at any specific time, the majority of primordial follicles are held in a dormant state, and when eventually recruited most will not reach the preovulatory stage but are destined to be removed through atresia at earlier stages of follicular development. Currently, clinical assessment is unable to assess the number of primordial follicles, or their rate of loss/activation. Knowledge of these aspects of ovarian function would be of value in a range of contexts, both clinical and social/personal, as well as being of great value in promoting our understanding of how reproductive lifespan is regulated.

Anti-Müllerian hormone (AMH), is now recognized as a principal regulator of early follicular recruitment from the primordial pool [4], [5], with AMH null mice demonstrating accelerated depletion of primordial follicle number and an almost three-fold increase in smaller growing follicles [6]. Furthermore this increase in number of growing follicles occurs despite lower serum follicle stimulating hormone (FSH) concentrations [7], suggesting that in the absence of AMH, follicles are more sensitive to FSH and progress through the early stages of follicular development. AMH is produced by small growing but not primordial follicles [8]–[10], although limited data suggest that serum AMH concentrations also correlate with primordial follicle number in humans [11] as in rodents [12]. The prepubertal endocrine environment is markedly different from the adult with low and non-cyclical gonadotropins: the relevance of this to AMH secretion is incompletely understood although follicle growth through the preantral stages and occasionally to early antral stages (i.e. across the full range of stages that secrete AMH) is observed in childhood [13]. A recent study has reported an increase in initial primordial follicular recruitment rates up to the age of puberty, and then a progressive decline to the menopause [14]. This suggests that AMH concentrations at any given age in both childhood and adulthood may mirror primordial follicular recruitment rates, rather than simply primordial follicle number. Consequently across the female lifespan, circulating AMH will potentially exhibit an initial increase followed by a non-linear decline as is well established for the primordial follicle pool [14]–[18].

In keeping with this, AMH concentrations in adults have been shown to decline with age [19], [20]. AMH concentrations are relatively stable across the menstrual cycle [21], [22] and also between cycles in the same woman [23]. Measurement of AMH is increasingly used in the prediction of ovarian response to superovulation [24]. Although large AMH cohort studies describing falling AMH concentrations in adult women (mostly from populations attending infertility clinics) have recently been published [25]–[28], the data for AMH concentrations in children have until recently been considerably more limited [29], [30]. To date no single study has examined AMH across the lifespan in healthy females. The aim of the current study is to produce a model of serum AMH in healthy females from conception to the menopause.

## Results

### The validated model

**Figure 1:**
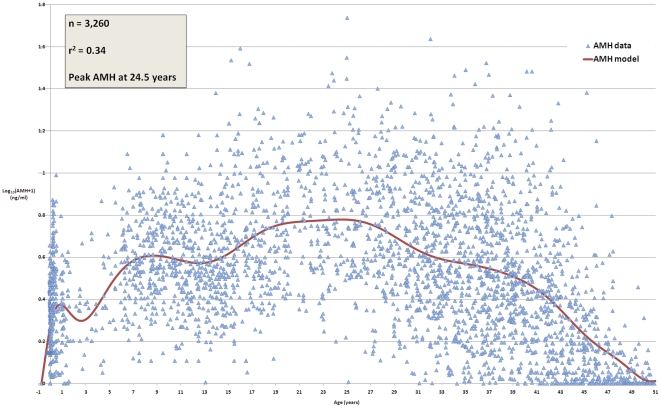
Serum AMH data. The red line is the model that best fits the 3,260 datapoints shown as triangles. The coefficient of determination, 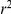, is 0.34, indicating that 34% of variation in serum AMH concentrations is due to age alone. Peak serum AMH is at 24.5 years.

Data from published studies and our own (Table 1) were used to derive the model (Figure 1). This included 3,260 data points across the age range from −0.3 (cord blood from preterm infants) to 54.3 years (Table 2). After 10-fold stratified cross-validation, the model with the highest coefficient of determination (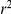) was a degree 20 polynomial of the form
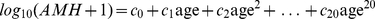
with coefficients 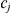 given in Table 3.

**Table 1:** Serum AMH data summary.

Ref.	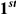 Author	Data	Assay	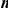	Average age	Age range	Det. lim.	Intra CV	Inter CV
[35]	Soto	Graph	IBC	58	30.3 (mean)	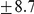 SD	0.10	5.3	8.7
[38]	Guibourdenche	Graph	IBC	192	NS	−0.3–1.0	0.30	5.3	8.7
[39]	Hudecova	Graph	IBC	64	46.3 (mean)	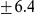 SD	0.70	12.3	12.3
[40]	Mulders	Graph	IBC	82	29.9	19.6–35.6	NS	5.0	8.0
[41]	Pastor	Graph	IBC	42	NS	18.0–50.0	0.10	5.3	7.8
[42]	Piltonen	Graph	IBC	44	31.6 (mean)	21.0–44.0	NS	5.1	6.6
[20]	van Rooij	Graph	IBC	162	NS	25.0–46.0	0.05	5.0	8.0
[43]	Laven	Graph	IBC	41	NS	20.0–36.0	0.05	5.0	8.0
[19]	de Vet	Graph	IBC	82	29.0	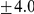 SD	0.05	5.0	8.0
[44]	Knauf	Graph	IBC	83	34.2 (mean)	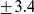 SD	0.03	11.0	11.0
[45]	Lee	Graph	IBC	225	NS	0.0–51.0	0.50	9.0	15.0
[36]	La Marca	Graph	IBC	24	44.0 (mean)	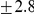 SD	0.24	5.0	8.0
[29]	Hagen	Graph	IBC	891	NS	0.0–68.0	0.03	7.8	11.6
[46]	van Beek	Graph	DSL	82	29.0	20.0–35.0	NS	5.0	15.0
[47]	Sanders	Graph	DSL	43	24.1 (mean)	0.1–51.0	0.01	NS	11.4
[34]	van Disseldorp	Graph	DSL	144	37.9 (mean)	25.0–46.0	0.03	11.0	11.0
[48]	Tehrani	Graph	DSL	267	27.1	16.0–44.0	0.01	5.2	9.1
[49]	Dorgan	Graph	DSL	204	44.7 (mean)	33.3–54.7	0.06	8.0	8.0
[30]	Ahmed	Raw	DSL	128	8.5	0.5–16.5	0.50	8.0	8.0
[25]	Nelson	Raw	DSL	441	36.1	21.9–47.8	0.03	3.4	8.6
	Total IBC			1,990	15.8	−0.3–68.0			
	Total DSL			1,309	35.4	0.2–54.7			
	Total 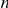			3,299	34.0	−0.3–68.0			
	**Censored total ** 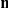			**3,260**	**28.3**	**−0.3–54.3**			

The references relate to the bibliography section of this paper. Age information is given as median and range, or as mean and standard devation (SD), depending on which form was reported in the referenced study. Detection limits are given in ng/ml. Intra- and inter-assay coefficients of variation (CV) are percentages. NS denotes not stated. For longitudinal studies – [19], [29], [40], [48] – we report the average age of participants at first measurement. The censored total excludes any values greater than 54.3 years (i.e. one standard deviation above the average age at menopause).

**Table 2:** Sample sizes at each age for the AMH model.

Age (yrs)	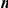	Age (yrs)	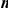	Age (yrs)	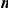	Age (yrs)	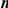	Age (yrs)	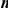	Age (yrs)	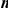
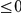	144										
0	277	10	61	20	21	30	77	40	79	50	25
1	43	11	69	21	37	31	77	41	72	51	18
2	12	12	65	22	27	32	101	42	80	52	19
3	12	13	61	23	35	33	74	43	66	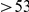	12
4	14	14	82	24	32	34	97	44	58		
5	14	15	61	25	50	35	84	45	59		
6	51	16	37	26	36	36	116	46	36		
7	60	17	64	27	55	37	98	47	37		
8	60	18	40	28	67	38	100	48	37		
9	58	19	40	29	45	39	86	49	23		

The 3,260 AMH datapoints described in Table 1, split into ages. 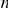 denotes the size of the subset of the data associated with an age in years.

**Table 3:** 10-fold cross-validation models.

	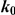	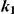	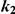	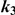	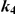	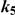	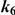	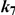	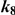	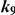	All data
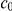	2.70e-01	2.67e-01	2.67e-01	2.67e-01	2.72e-01	2.69e-01	2.67e-01	2.71e-01	2.70e-01	2.68e-01	2.69e-01
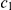	2.78e-01	2.91e-01	2.91e-01	2.75e-01	2.84e-01	2.83e-01	2.62e-01	2.50e-01	2.79e-01	2.69e-01	2.77e-01
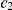	-1.92e-01	-1.90e-01	-1.90e-01	-1.91e-01	-1.98e-01	-1.78e-01	-1.87e-01	-1.91e-01	-1.86e-01	-1.74e-01	-1.88e-01
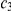	-3.21e-02	-4.82e-02	-4.82e-02	-3.27e-02	-3.59e-02	-3.42e-02	-1.54e-02	-2.93e-04	-2.95e-02	-1.66e-02	-2.99e-02
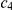	7.17e-02	8.45e-02	8.45e-02	7.22e-02	7.92e-02	6.39e-02	5.99e-02	4.72e-02	6.64e-02	5.14e-02	6.85e-02
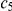	-3.13e-02	-3.62e-02	-3.62e-02	-3.14e-02	-3.52e-02	-2.64e-02	-2.78e-02	-2.23e-02	-2.86e-02	-2.27e-02	-3.00e-02
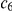	7.56e-03	8.71e-03	8.71e-03	7.57e-03	8.66e-03	6.14e-03	6.98e-03	5.53e-03	6.85e-03	5.55e-03	7.25e-03
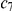	-1.21e-03	-1.38e-03	-1.38e-03	-1.20e-03	-1.40e-03	-9.56e-04	-1.15e-03	-8.98e-04	-1.09e-03	-9.02e-04	-1.16e-03
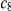	1.38e-04	1.57e-04	1.57e-04	1.37e-04	1.61e-04	1.07e-04	1.35e-04	1.03e-04	1.23e-04	1.05e-04	1.33e-04
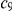	-1.17e-05	-1.31e-05	-1.31e-05	-1.15e-05	-1.36e-05	-8.90e-06	-1.16e-05	-8.83e-06	-1.04e-05	-9.08e-06	-1.12e-05
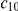	7.50e-07	8.35e-07	8.35e-07	7.33e-07	8.75e-07	5.66e-07	7.61e-07	5.72e-07	6.64e-07	5.97e-07	7.20e-07
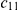	-3.71e-08	-4.08e-08	-4.08e-08	-3.60e-08	-4.31e-08	-2.78e-08	-3.83e-08	-2.85e-08	-3.28e-08	-3.02e-08	-3.56e-08
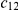	1.43e-09	1.55e-09	1.55e-09	1.38e-09	1.65e-09	1.06e-09	1.49e-09	1.10e-09	1.26e-09	1.19e-09	1.37e-09
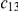	-4.27e-11	-4.57e-11	-4.57e-11	-4.09e-11	-4.89e-11	-3.16e-11	-4.50e-11	-3.30e-11	-3.78e-11	-3.63e-11	-4.08e-11
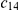	9.89e-13	1.05e-12	1.05e-12	9.41e-13	1.12e-12	7.31e-13	1.05e-12	7.68e-13	8.76e-13	8.56e-13	9.44e-13
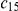	-1.76e-14	-1.83e-14	-1.83e-14	-1.66e-14	-1.98e-14	-1.29e-14	-1.88e-14	-1.37e-14	-1.56e-14	-1.55e-14	-1.67e-14
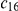	2.34e-16	2.41e-16	2.41e-16	2.21e-16	2.61e-16	1.72e-16	2.52e-16	1.83e-16	2.09e-16	2.10e-16	2.23e-16
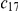	-2.27e-18	-2.31e-18	-2.31e-18	-2.13e-18	-2.51e-18	-1.67e-18	-2.46e-18	-1.78e-18	-2.03e-18	-2.06e-18	-2.16e-18
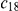	1.51e-20	1.52e-20	1.52e-20	1.41e-20	1.65e-20	1.11e-20	1.64e-20	1.19e-20	1.36e-20	1.39e-20	1.43e-20
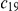	-6.16e-23	-6.12e-23	-6.12e-23	-5.74e-23	-6.66e-23	-4.54e-23	-6.74e-23	-4.84e-23	-5.55e-23	-5.75e-23	-5.83e-23
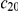	1.16e-25	1.14e-25	1.14e-25	1.08e-25	1.24e-25	8.56e-26	1.27e-25	9.14e-26	1.05e-25	1.10e-25	1.10e-25

The columns are the 21 coefficients 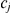 for the degree 20 polynomial returned by TableCurve2D that gave the highest 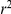 for the dataset under consideration. For fold 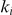, the dataset consisted of all points except the 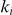 test data. After validation of the degree 20 polynomial (Table 3), its coefficients were calculated for the entire dataset (final column). We report this model as our validated model of serum AMH from conception to menopause.

This model has an 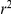 of 0.34, indicating that roughly one third of variation in AMH concentrations is due to age alone, with the remaining two thirds of the variation being due to other factors. Serum AMH peaks at 24.5 years on average, with concentrations decreasing shortly after birth, and again between eight and twelve years of age.

### Validation

**Figure 2:**
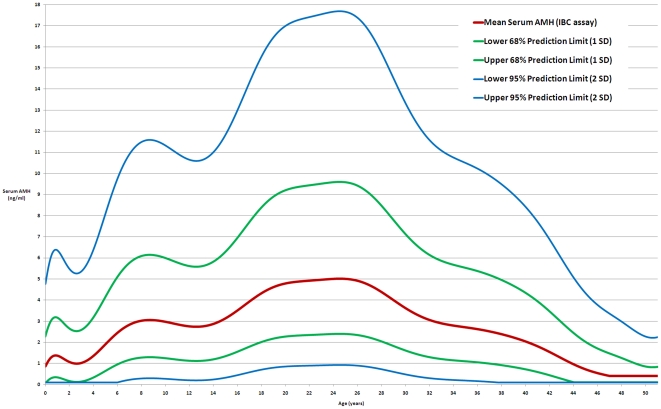
The normal range for serum AMH in girls and women. The red line is the log-unadjusted validated AMH model using IBC assay values. The blue and green lines are the 68% and 95% prediction limits for the model (plus and minus one and two standard deviations respectively).

For each of the 10 folds of the validation process, the highest ranked of 215 models was a polynomial similar to the model reported above: in every case the returned coefficients, 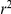 and peaks were similar (Table 4). Moreover, the average training error was within 1% of the average cross-validation estimate of the prediction error across 10 folds (Table 4). We therefore consider that the model derived for all 3,260 datapoints generalises well to unseen data, and hence report this as a validated model for serum AMH concentrations in the normal female population (Figure 2).

**Table 4:** 10-fold cross-validation results.

	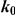	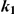	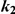	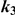	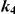	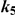	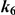	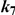	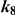	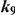	Mean
MSE train	0.069	0.068	0.067	0.069	0.069	0.069	0.069	0.069	0.069	0.068	**0.069**
MSE test	0.064	0.070	0.080	0.065	0.068	0.069	0.067	0.068	0.064	0.078	**0.069**
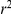	0.338	0.338	0.341	0.352	0.343	0.339	0.341	0.340	0.337	0.345	**0.341**
Age at peak AMH	25.1	21.7	21.5	22.0	25.5	24.5	24.6	24.5	24.1	25.0	**23.8**

The mean squared error (MSE) for the model with highest 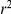 is given for both the training set (90% of the dataset) and the test set (the remaining 10%) for each of the ten folds. Since – both for individual folds and on average – the errors are similar, we consider the model to be validated. The 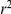 and peak ages are for the highest ranked model returned by TableCurve2D for each fold.

## Discussion

We have generated the first validated model of serum AMH in the healthy human population from conception to menopausal ages. Our model shows that serum AMH concentrations peak at age 24.5 years for the average case, and suggests that two thirds of the variation in AMH concentrations for healthy females is due to factors other than age.

We have shown that serum AMH falls shortly after birth, with concentrations only increasing again after about two years of age. This feature is in line with evidence of a mini-puberty seen in neonatal girls [31], [32], although more clearly characterized in boys [33], and with a recent longitudinal study of AMH in female neonatal blood [29] (the data from which were included in our study). Our model also shows that serum AMH concentration falls between the ages of eight and twelve, before rising to a peak in the mid-twenties. This fall may be an artefact of our model derivation process rather than a true reproductive biological event. As the fall coincides with the initial increases in gonadotropin concentrations of early puberty, it is possible that it reflects changes in the proportions of follicles at different stages of growth with increasing numbers progressing to antral stages rather than becoming atretic early on [3]. AMH is produced by early growing follicles at all stages up to the early antral stage [8] but it is unknown which follicle class contributes most to circulating concentrations. The rising granulosa cell mass (and thus AMH production per follicle) will be balanced by progressively declining numbers of follicles at each stage of growth [2], [3].

Our incomplete understanding of how early follicle recruitment and growth are regulated means that any such interpretation is speculative but our results both suggest this avenue of future research, and give useful indications of effect sizes and ages for the design of such investigations.

The increase in AMH during the postnatal period, supported by a recent longitudinal analysis in the first 3 months of life [29], is likely to be analogous to the well established transient rise in testosterone and inhibin B in boys at that time [33]. This is likely to reflect the relatively high gonadotrophin concentrations that are present which will support more advanced follicle development than occurs in the remainder of the prepubertal period. Consistent with this, the ovary shows follicle growth to the early antral stage from birth [13]. The continuing rise in AMH through childhood is striking, and parallels rising follicle growth initiation from the very large pool at these ages [14]. The rising AMH production would therefore act to limit follicle growth activation [5], [6], thus a point of inflection when follicle recruitment starts to slow, and which is followed by a decline in AMH concentrations is predictable: our data demonstrate the age at which this occurs.

Our observed increase in AMH concentrations beyond the age of 12 years, is in contrast to the recent analysis by Hagen *et al*. which suggested in a cross-sectional study that AMH did not change from age 8 to age 25 or indeed relative to pubertal stage [29]. Censoring our own data between these two ages would also suggest that AMH does not vary markedly, however, this would lose the power of the broader picture afforded by the entire age range analysis performed here. The lack of an increase beyond age 12 as suggested by Hagen *et al*., is in marked contrast to current and previous studies, which have all suggested a peak in early adulthood. A recent analysis of 9,601 infertility patients [25] and a smaller study of 82 healthy subjects both report that AMH concentrations decrease after age 20 [19]. Although two studies have reported peak AMH at age 31–33 [34], [35], these were substantially smaller incorporating 144 and 58 subjects and the minimum age was 14 years and 25 years respectively. This smaller sample size and lack of data from younger (especially neonatal) subjects may have skewed the reported peaks towards a higher age.

We recognize that although our study has a number of limitations – including the accuracy of the health status of subjects in the studies used and the dependence on accurate graphical presentation of published results – it also has considerable strengths. The dataset was derived from over 3,500 subjects with ages ranging from minus 0.3 years to 68 years, the model was then validated using standard mathematical techniques, with very good generalisation to unseen data for each of the 10 cross-validation steps. We did not make any presumptions regarding the optimal fitting model, yet the optimal model exhibited a non-linear decline in AMH in adult life consistent with previous cross-sectional and longitudinal studies [25], [34], [36], [37], and the minipuberty seen in neonatal girls [29], [31], [32]. This validated model can therefore be used to accurately interpret concentrations of serum AMH in females across the lifespan. The model is based entirely on cross-sectional data (the small amount of longitudinal data was treated as if cross-sectional). Further longitudinal validation in similarly large populations will provide additional confirmation.

It should be noted that our methodological choices have no effect on the qualitative nature of our results. If we convert to DSL values instead of IBC, do not add one to the log-adjusted values for ease of exposition, do not censor at age 54.3, allow models with more parameters (or restrict to models with slightly fewer parameters), use 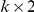 or bootstrap cross-validation, we still obtain a validated model with similar peaks, 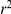 and level of generalisation to unseen data. The current introduction of the GenII AMH assay (Beckman Coulter) uses the same standards as the IBC assay, thus we anticipate that the normative model presented (Figure 2) will be valid for values obtained using that system.

This comprehensive statistical analysis of 3,260 healthy infants, children and women has facilitated the first validated normative model of age related circulating AMH from conception to the menopause. The model provides a means for interpretation of how an individual's serum AMH concentration compares with population norms.

## Materials and Methods

### Data acquisition

Studies involving serum AMH measurements of human females were identified by performing PubMed and Medline searches and searching individual journals (including Menopause, Fertility and Sterility, Human Reproduction and the Journal of Clinical Endocrinology and Metabolism) using the search terms AMH, Müllerian inhibiting substance, ovarian reserve and polycystic ovarian syndrome. The references of included studies (Table One; [19], [20], [29], [34]–[36], [38]–[49]) were checked to identify further relevant studies to be processed. Data was selected for this analysis only for subjects who were not known to be infertile, or have an identified chronic illness. Hence all subjects were either in control groups from controlled studies or from prospective studies of the healthy population. Any data from subjects with a chronic disease or undergoing infertility assessment or investigation were excluded from the study. In the main, the data was from pre-menopausal women. In three studies the menopausal status of the women was not stated [45], [47], [49]. Data from fetal blood (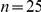) and cord blood (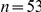) of infants [38] were included. Longitudinal data – from [19], [29], [40], [48] – were recorded as cross-sectional values. The data were extracted from graphs using Plot Digitizer software [50] to convert datapoints on the graphs into numerical data. Repeated datapoints were isolated by requiring that the acquired dataset matched the descriptive statistics provided in the supporting paper (Table One).

We combined the resulting dataset with two sets of raw Scottish data. The first consisted of individual serum AMH measurements (n = 441, median age 36.1 years, max. age 47.8, min age 21.9) from a cohort of women whose partners were known to have severe male factor infertility requiring ICSI, and where no other female cause of infertility had been identified [25]. Individual patient serum AMH measurements were undertaken between July 2006 and October 2009 in the biochemical laboratories of the University of Glasgow, the Glasgow Centre for Reproductive Medicine and the Glasgow Royal Infirmary. All three facilities were providing centralised AMH testing for infertility clinics within the United Kingdom. Ethical approval for studies involving these data was obtained from NHS Scotland. Subjects were informed that data may be analysed anonymously. Individual ethical approval has not been taken for studies involving this data, as it is routine anonymous clinical data which is covered by the general UK National Health Service ethics for analysis of routine biochemical data, provided it is anonymous, The second dataset was supplied by Ahmed et al. [30] and consists of 128 measurements taken from subjects aged 0.5–16.5 years.

Serum AMH values were standardised to give AMH measurements in ng/ml using the conversion formula 1 pmol/l = 7.143 ng/ml.

The resulting data were considered separately depending on the assay used to obtain serum AMH values. The first dataset (n = 1,990, median age 15.8 years, max. age 68.0, min age −0.3) came from those studies in which the serum concentrations of AMH were determined using enzyme-linked immunoassay kits IBC (Immunotech Beckman Coulter Company, France). The second dataset (n = 1,309, median age 35.4 years, max. age 54.7, min age 0.2) came from studies in which the enzyme-immunometric assay Active MIS/AMH ELISA kits DSL (Diagnostic Systems Laboratories Inc., TX, USA) were used. We converted the DSL data into IBC values using the conversion formula
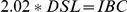
which has a reported 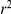 of 0.85 [51], and censored 39 datapoints over 54.3 years (mean age at menopause plus one standard deviation [52].) The resulting dataset consists of 3,260 serum AMH concentrations at known ages, and approximates circulating AMH concentrations in the healthy population from conception to menopause.

### Data analysis

Stratified 10-fold cross-validation was performed using standard techniques [53]. The dataset was split into 10 distinct subsets, 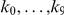, of nearly equal size, each with similar mean, median, minimum and maximum. For fold 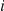, 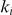 was retained as test data, with the remaining 90% of the data used for training purposes. For each training set we added zero AMH values at conception, in order to force models through the only known AMH concentration at any age. Since variability increases with AMH concentration, we log-adjusted the data (after adding one to each value so that zero AMH on a chart represents zero serum AMH). All data were analysed as cross-sectional values (i.e. longitudinal patterns were not considered). We then fitted 215 mathematical models to the 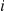-th test data using TableCurve-2D (Systat Software Inc., San Jose, California, USA), and ranked the results by coefficient of determination, 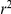. Each model defines a generic type of curve and has parameters which, when instantiated gives a specific curve of that type. For each model we calculated values for the parameters that maximise the 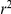 coefficient. The Levenberg-Marquardt non-linear curve-fitting algorithm was used throughout, with convergence to 5 significant figures after a maximum of 1,000 iterations. The highest ranked model was chosen as the best model for the test data, and the mean square error and 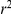 were calculated after removing the artificial zero values at conception.

For validation purposes, the mean square error of the 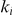 data for the 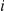-th model was calculated and compared to the mean square error of training data for the same model. In other words, the cross-validation estimate of the prediction error of the model was compared to the training error of the model. Training error is expected to be underestimated (due to overfitting of the chosen data) and the cross-validation estimate of the prediction error is expected to be overestimated (due to underfitting of unseen data). We consider a model to be validated if the differences between these errors is small.

For each fold the highest ranked model was a degree 20 polynomial of the form
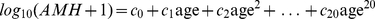
Hence, after validation (Tables 3 and 4), the 21 parameters for this model were derived for the entire dataset (n = 3,260), again using TableCurve2D. The resulting model is reported as our validated model (Figure 2).
